# Ultrasonic Welding of PEEK Plates with CF Fabric Reinforcement—The Optimization of the Process by Neural Network Simulation

**DOI:** 10.3390/ma16052115

**Published:** 2023-03-06

**Authors:** Vladislav O. Alexenko, Sergey V. Panin, Dmitry Yu. Stepanov, Anton V. Byakov, Alexey A. Bogdanov, Dmitry G. Buslovich, Konstantin S. Panin, Defang Tian

**Affiliations:** 1 Laboratory of Mechanics of Polymer Composite Materials, Institute of Strength Physics and Materials Science of Siberian Branch of Russian Academy of Sciences, 634055 Tomsk, Russia; 2 Department of Materials Science, Engineering School of Advanced Manufacturing Technologies, National Research Tomsk Polytechnic University, 634050 Tomsk, Russia; 3 Laboratory of Nanobioengineering, Institute of Strength Physics and Materials Science of Siberian Branch of Russian Academy of Sciences, 634055 Tomsk, Russia; 4 Department of Chemical Physics, Institute for Laser and Plasma Technologies, National Research Nuclear University MEPhI, 115409 Moscow, Russia

**Keywords:** machine learning, neural network simulation, carbon fiber fabric, ultrasonic welding, lap joint, PEEK, prepreg, interface, adhesion, structural integrity

## Abstract

The optimal mode for ultrasonic welding (USW) of the “PEEK–ED (PEEK)–prepreg (PEI impregnated CF fabric)–ED (PEEK)–PEEK” lap joint was determined by artificial neural network (ANN) simulation, based on the sample of the experimental data expanded with the expert data set. The experimental verification of the simulation results showed that mode 10 (t = 900 ms, P = 1.7 atm, τ = 2000 ms) ensured the high strength properties and preservation of the structural integrity of the carbon fiber fabric (CFF). Additionally, it showed that the “PEEK–CFF prepreg–PEEK” USW lap joint could be fabricated by the “multi-spot” USW method with the optimal mode 10, which can resist the load per cycle of 50 MPa (the bottom HCF level). The USW mode, determined by ANN simulation for the neat PEEK adherends, did not provide joining both particulate and laminated composite adherends with the CFF prepreg reinforcement. The USW lap joints could be formed when the USW durations (*t*) were significantly increased up to 1200 and 1600 ms, respectively. In this case, the elastic energy is transferred more efficiently to the welding zone through the upper adherend.

## 1. Introduction

Laminated polymer composites reinforced with continuous carbon fibers (CFs), so- called laminates, are widely applied in the aerospace and other high-tech industries. Zhang et al. [1] reviewed many global industrial applications of laminated composites in aerospace, wind energy, machine building, high pressure vessels, civil engineering, etc. Typically, thermoset resins were used to fabricate such materials, but thermoplastic ones have actively replaced them nowadays [2,3]. As evidence, a concise review of out-of-autoclave prepregs is presented in [4]. In addition, some new methods to produce CF-reinforced composites, including additive manufacturing, are being actively developed [5,6,7,8].

To this end, one of the important challenges is joining the CF-reinforced composites [9,10,11,12]. Based on the thermoplastic binders, the latter is solved by ultrasonic welding (USW) [13,14,15,16].

When developing the USW procedures for laminates, the influence of various technological parameters on the structure and functional properties of such joints is investigated. The research studies on such phenomena and the issues concerning optimization of the USW parameters include examining both heat generation and its transfer [17,18,19], the transmission of ultrasonic (US) vibrations [20,21], applied power and sonotrode displacement [22], adding a consolidator to a welding setup [23], effects of heating and shapes of energy directors (ED) [24,25,26], the possibility of USW joining of thermoplastics with thermosets [27]; principles of controlling the USW process [28]; the material crystallinity in a fusion zone [29]; both distribution and evolution of temperature fields [30]; the strain behavior under various test conditions [31], etc.

Even though few knowledge gaps remain in the USW of laminates, great interest in this topic still exists among researchers. In particular, two important areas should be noted: (i) the development of USW procedures for the particulate composites rather than the layered ones [32], and (ii) the production of the prepregs [33] or the US-assisted fabrication of multilayered CFRC [34].

Concerning USW joining the CFRC, some relevant reports of the scientific groups should be pointed out [35,36,37,38]. In addition, papers [39,40,41] are focused on developing repairing procedures. In terms of US-assisted prepreg fabrication, the automated placement of the CFs should be considered a very promising issue to discuss [42,43]. An alternative to this method is consolidating a composite pipe by in situ USW processing of a thermoplastic matrix composite tape [44]. One of the key issues to be solved regarding the optimization of the USW parameters is to consider both the viscosity and fluidity of the thermoplastics, as well as the impregnation of the CFFs [45].

Bonmatin et al. proposed an approach to the USW of laminates that included two layers of polyetherimide (PEI) between two polyetheretherketone (PEEK)/CF adherends, in addition to the PEI-based ED [46]. Considering the development of additive manufacturing of both polymers and composites, Khatri et al. investigated the efficiency of the USW of 3D-printed parts from both neat PEEK and its CF-reinforced composites [47]. It was suggested that such USW procedures, developed for the 3D-printed high-performance thermoplastics, might be implemented for manufacturing both non- and load-bearing structures.

It should also be noted that there is a distinction between continuous and multi-spot USW methods [48] since their clamping and heat input patterns are different. For this reason, their optimal parameters may differ significantly.

Based on the above, optimization of the joint formation conditions is a vector approximation task due to the variety of possible USW modes and their numerous parameters (for example, the process duration, clamping pressure [49,50,51], amplitude and frequency of US vibrations, etc.) and deployed equipment. Therefore, among the most effective approaches to its solution are approaches based on the methods of ANN.

In the previous study [52], the authors developed a methodology for determining the optimal USW parameters. The methodology included the sequential solution of a set of tasks. Among them were (i) planning an experiment; (ii) determining and analysis of the experimental data, including the selection of the most suitable functional properties; (iii) the USW process simulation; (iv) searching for the optimal parameters; (v) the experimental verification of the adopted model. The Taguchi method was applied to plan the experiment and analyze its results. At the same time, the simulation algorithms were based on ANN with varying parameters (the number of neurons and the type of activation functions, as well as training methods). As part of the experimental verification of the adopted model, the samples of the USW lap joints were fabricated, containing a PEEK-based prepreg with the unidirectional continuous CFs as a central layer. It was found that damage to the unidirectional CF tapes might occur when the prepreg is melted. The results revealed the problem of the USW lap joint formation with the prepreg from the CFF impregnated via a thermoplastic solution. It has stimulated further research on improving the ANN approach for the USW process simulation.

Thereby, the aim of this study is to find out the optimal USW parameters for the lap joints with the neat PEEK adherends and the PEI-impregnated CFF prepreg as the central layer via a simulation process carried out with ANNs. The paper is structured as follows. Section 2 describes both the implemented techniques and deployed equipment. Section 3 briefly characterizes the prepreg structure. Section 4 presents the results of the mechanical testing and analysis of the lap joints’ structure. Section 5 includes the simulation results aimed at determining the optimal USW parameters. Finally, Section 6 and Section 7 discuss the results and their prospects.

## 2. Materials and Methods

The “770PF” PEEK powder (Zeepeek, Changchun, Jilin Province, China) was used as a feedstock for manufacturing plates (adherends) with dimensions of 100 mm × 20 mm × 2.2 mm using the “RR/TSMP” injection molding machine (Ray-Ran Test Equipment Ltd., Nuneaton, UK). The mold heating temperature range was 200–205 °C; the powder feeder (hopper) was heated to 395 °C.

**Figure 1:**
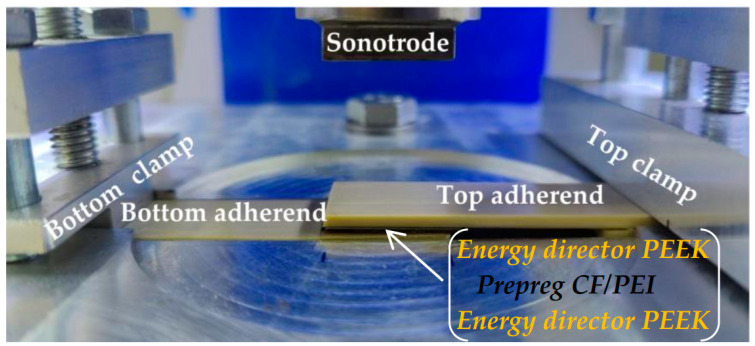
The USW facility.

The USW lap joints were formed with the ED from a commercially available PEEK film Aptiv 2000 250 µm thick (Victrex, Lancashire, UK) cut into 22 × 22 mm square pieces. Before the USW process, the EDs were placed between the PEEK adherends while the prepreg was located in the center of the joints (Figure 1). A description of the prepreg fabrication technique is described in Section 3. “UZPS-7” ultrasonic welding machine was explored (SpetsmashSonic LLC, Voronezh, Russia). The plates to be welded were placed in a fixing clamp to prevent their mutual movement. A sonotrode with square geometry and 20 × 20 mm dimension was used. The USW lap joints included five layers; two outer neat PEEK adherends, the central CFF prepreg layer, and two intermediate ED (Figure 1). The USW lap joint thinning was measured with a micrometer.

It should be noted that the USW machine used for this study provided the possibility of varying the USW duration and the clamping pressure. However, the US oscillation amplitude was constant at 10 μm. The US vibration frequency of 20 kHz was also not varied.

In the experimental design, the following levels of the USW parameters (the simulation factors) and their possible variation ranges were preset (Table 1):

1. The USW duration (*t*) range was set as 600, 850, and 1150 ms since it was not possible to join the PEEK plates at lower *t* values (since the heat input was low to initiate the melting of the PEEK). However, the prepreg could be seriously damaged at higher *t* levels, resulting in highly faulty USW lap joints.
2. Ranges of the clamping pressure (*P*) and its (holding) duration after applying US vibrations (*τ*) were determined based on the technical characteristics of the USW machine and by visual control of the USW lap joints.

The tensile tests of the USW lap joints were performed according to ASTM D5868. The tests were carried out with an “Instron 5582” electromechanical tensile testing machine (Instron, Norwood, MA, USA). The cross-head speed was 13 mm/min. To minimize misalignment during the tests, gaskets made of identical-thickness PEEK plates were installed in the wedge grips.

The structure of cross-sections of the USW lap joints was analyzed with “Neophot 2” optical microscope (Carl Zeiss, Jena, Germany) after the tensile tests.

**Figure 2:**
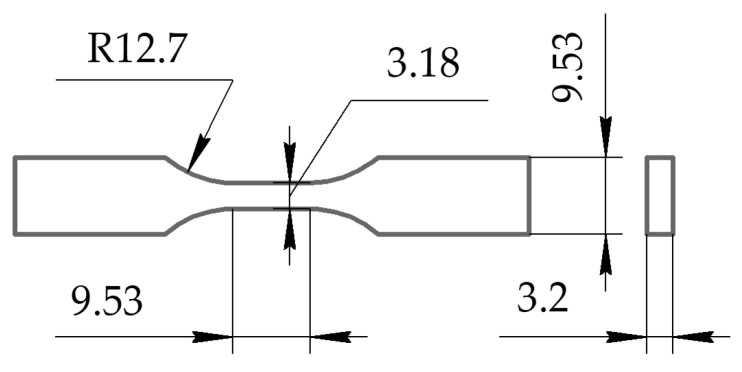
A scheme of the specimens for the static tension and fatigue tests (all dimensions in mm).

As mentioned above, both continuous and multi-spot USW modes could be applied. For a qualitative assessment of suitability of the optimal USW parameters, the samples for the fatigue tests were fabricated by using the PEEK plates, the prepreg from the PEI-impregnated CFF, and the PEEK film EDs (like the data described below in Section 4). All welded elements had the same width and length. The USW process was carried out by joining the details at three spots. Then, the “dog-bone” samples (Figure 2) were cut with a milling machine. The fatigue tests were carried out in the quasi-static axial tension mode using “Biss Nano” universal servo-hydraulic machine with “Biss Bi-06-103 15 kN” load cell (ITW India Private Limited (BISS), Bangalore, India) at a cross-head speed of 1 mm/min and a load sampling rate of 100 Hz. To characterize the strain development quantitatively, the non-contact digital image correlation (DIC) method was applied to calculate the parameters of the mechanical hysteresis loops.

The strain values were sampled at a frequency of 5 Hz and then evaluated by the DIC method. For drawing the strain fields, a speckle pattern was deposited on the sample surfaces, which was captured with “Point Gray Grasshopper 50S5M” digital camera (Point Gray Research^®^ Inc., Vancouver, BC, Canada) and “Sony^®^ ICX625 2/3” CCD matrix 2448 × 2048 (a resolution of 5 megapixels, matrix dimensions of 8.4 × 7.0 mm, and pixel sizes of 3.45 × 3.45 µm). The required surface illumination level was provided with “Jinbei EF-100 LEDSun Light” LED illuminator (Shanghai Jinbei Photographic Equipments Co., Ltd., Shanghai, China). The analysis of the sample surface images and a drawing of the strain fields were conducted using VIC 2D 2009 software (Correlated Solutions Inc., Columbia, SC, USA).

To assess the fatigue properties of the USW lap joints, cyclic loading of the samples was carried out in the “tensile–tensile” mode with the load control, a sinusoidal shape of the loading signal, and a zero-asymmetry cycle (the *R* ratio of 0). The loading frequency was 1 Hz with a periodic deceleration down to 0.05 Hz (measuring cycle) for photographing the sample surfaces with the DIC. For implementing the high-cycle fatigue (HCF) mode, the maximum stress per cycle *σ*_max_ was chosen, ~50 MPa, which was below the yield point (0.8 · *σ*_0.2_). For the low-cycle fatigue (LCF) mode, a *σ*_max_ value of 55 MPa was preset, which was 0.85 of the ultimate tensile strength level.

## 3. Fabrication of the Impregnated Prepregs

Our previous study was conducted on the variation of the PEEK binder contents in a CFF-based prepreg [53]. It was shown that the molten thermoplastic in the prepreg intensively extruded from the fusion zone during the USW process, tearing the CFF if the thickness of the latter was significantly lower than that of the prepreg (at PEEK/CF weight ratios of 50/50 and above). In doing so, the prepreg thickness was to be minimized, and the entire volume of the CFF had to be impregnated with a thermoplastic binder.

Since the prepreg was fabricated under laboratory conditions, additional studies were carried out on impregnating the CFF with thermoplastic solutions without using strong acids. For this reason, the option of dissolving PEEK was not considered. However, assuming that a chemical bond was formed between the adherends and the PEEK film ED from the USW process, polyetherimide (PEI) and polyethersulfone (PES) were taken as options. First, the PEI and PES were dissolved in N,N-Dimethylformamide (C_3_H_7_NO). Then, the fragments of the CFF were soaked in the solution for one hour, followed by evaporation of the organic solvent on the “IKA C-MAG HP 4” heater (IKA^®^-Werke GmbH & Co. KG, Staufen im Breisgau, Germany). The latter was conducted under ventilation conditions to guarantee the removal of volatiles.

### The Prepregs Impregnated with PES and PEI

The prepreg was fabricated as follows. In order to remove the technological (epoxy) sizing agent from the surfaces, the CFF was annealed at 500 °C for 30 min. Then, it was impregnated with the dissolved PES. Finally, the solvent was evaporated in an oven at 120 °C.

**Figure 3:**
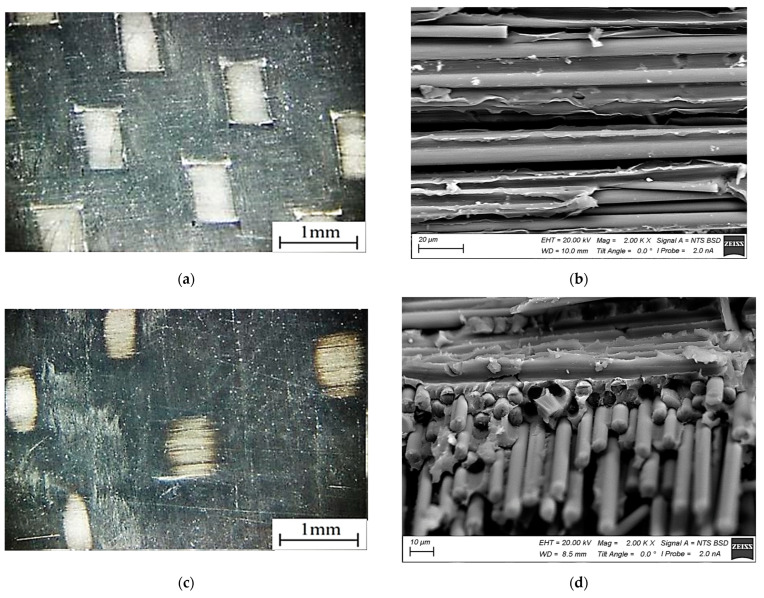
The general views from the surfaces (**a**,**c**), as well as the SEM micrographs of the cross-sections (**b**,**d**) of the prepregs from the annealed CFF impregnated with the PES (**a**,**b**) and the PEI (**c**,**d**).

As a result, the impregnated CFF thickness was ~500 µm. After that, it was subjected to compression molding in order to reduce the thickness to that of the initial CFF of 230–250 µm. In the prepreg, the ratio of the components was 66 wt.% CFF/34 wt.% PES. Finally, the thickness of the prepreg (the CFF impregnated with the polymer) was ~250 µm (Figure 3a,b). 

The prepreg from the PEI-impregnated CFF was made in a similar way. As a result, the component ratio and the final prepreg thickness were also identical (66 wt.% CFF/34 wt.% PEI and ~250 µm, respectively). Its general view from the surface and SEM micrographs of the cross-section is shown in Figure 3c,d.

Next, the prepregs were compared by their effect on the strength properties of the USW lap joints, obtained with the following parameters preset based on our previous results [52]: a USW duration of 1000 ms, a clamping pressure 1.5 atm, and a clamping duration after US vibrations of 5000 ms. The results of the tensile tests are presented in Table 2. 

Additionally, the USW joint thinning was evaluated with a micrometer. In the case of the PES-impregnated prepreg, it was ~410 µm, while it was reduced to ~290 µm for the PEI one (Table 2). According to the visual inspection results for the cross-sections of the USW lap joints, the polymer melting occurred both in the prepreg and partially in the EDs during the USW process. However, the PEI and PES melting points were lower than that of the PEEK. In addition, the presence of 66 % CFF increased the prepreg’s integral thermal conductivity and heat capacity. In the PES case, its more intensive extrusion occurred due to the higher melt flow index, which could be accompanied by damage to the CFF. Based on these data, only PEI-impregnated prepreg was used further.

## 4. Mechanical Test Results

**Figure 4:**
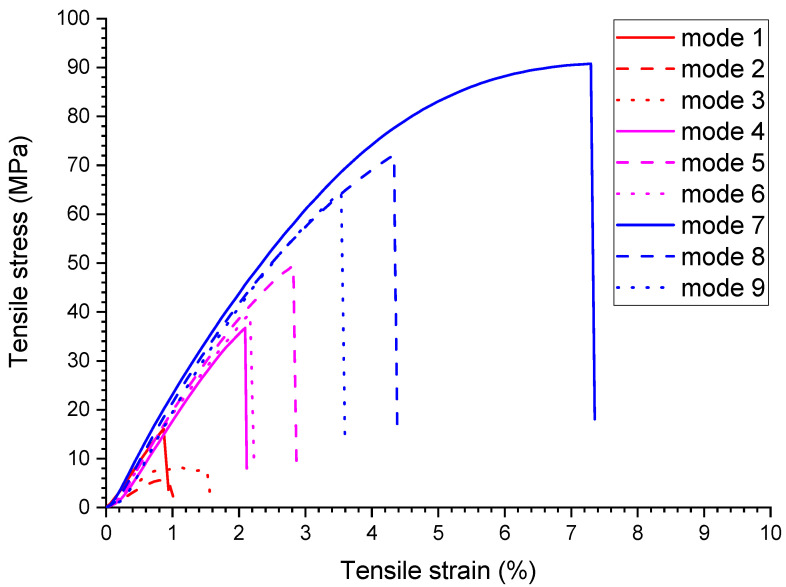
The strain–stress diagrams for the USW lap joints obtained using modes 1–9.

The strain–stress diagrams for the USW lap joints are shown in Figure 4, and the results of their mechanical tests are presented in Table 3. 

Additionally, the USW joint thinning values, measured with the micrometer, are reported. The primary data processing was carried out by the Taguchi method. The results are presented in the Supplementary Materials (Tables S1–S5, Figure S1).

**Figure 5:**
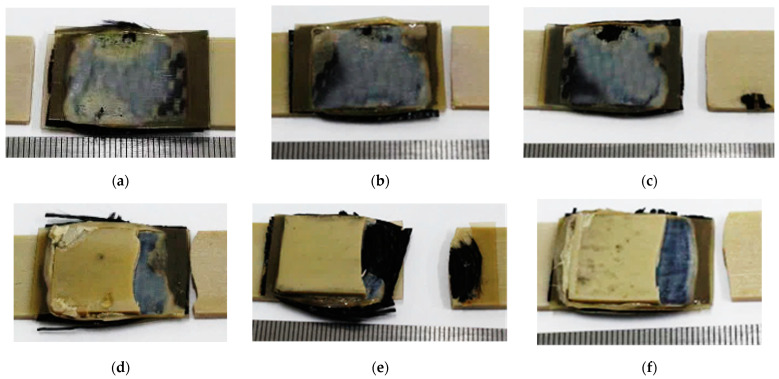

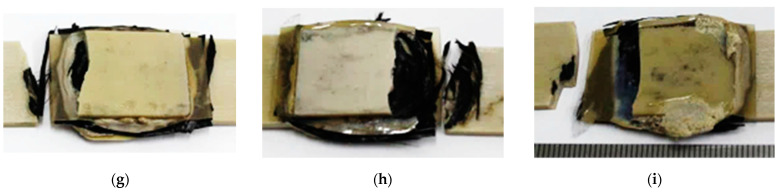
General view photos of the fractured USW lap joints after the tensile tests ((**a**–**i**), for modes 1–9, respectively).

**Figure 6:**
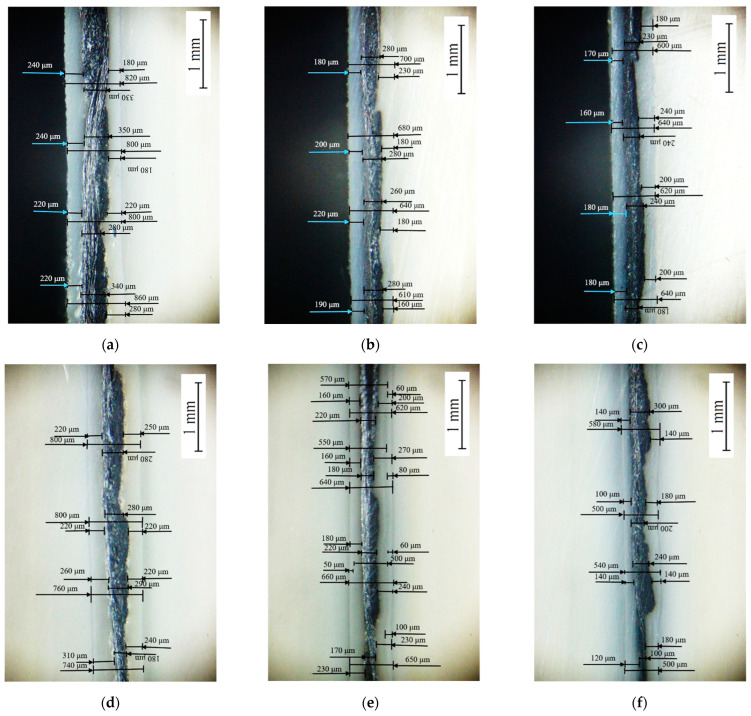

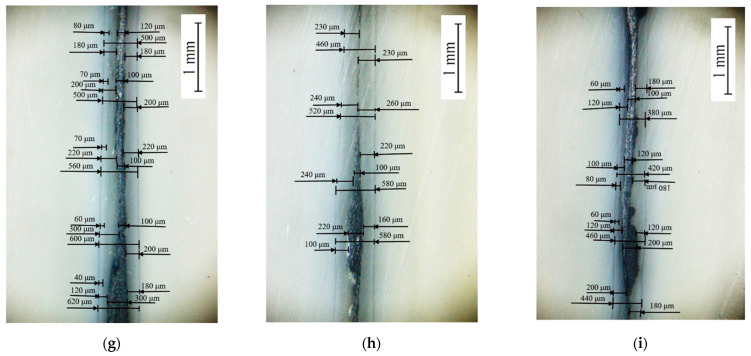
General-view photos of the fractured USW lap joints after the tensile tests ((**a**–**i**), for modes 1–9, respectively).

After the tensile tests, the failure patterns of the USW lap joints were analyzed (Figure 5 and Figure 6). Table 4 presents distances between the joined PEEK plates and the CFF thicknesses, determined by the analysis of the optical images shown in Figure 6. These data were used to interpret the revealed patterns.

Note that the authors failed to solve the macrobending problem caused by the double thickness of the central part of the specimens [54] in the tensile tests of the USW lap joints. This issue was investigated numerically in the previous study [55]. Macrobending greatly determined the resistance of the specimens to failure and contributed to minimizing the influence of the interlayer shear mechanism. For the same reason, the ultimate tensile strength σ_UTS_ parameter was used as a strength characteristic of the lap joints rather than the lap shear strength (LSS) one. Its impact was minimized by ANN simulation and optimization of the USW parameters (Section 5).

When forming the USW lap joint using mode 1 (*t* = 600 ms, *P* = 1.5 atm; Figure 5a), the EDs did not (or partly) melt locally. This fact reflected a non-uniform distribution of the temperature field (and, accordingly, the lap joint structure). In the prepreg, the CFF was impregnated with the PEI, while the EDs were the PEEK film. Thus, their thickness decreased slightly to 180–240 µm relative to the initial thickness of 250 µm (Figure 6a). The EDs’ thicknesses differed to the left and right of the prepreg, but they did not exceed 90 µm (Table 3) at σ_UTS_ = 16.1 MPa.

The subsequent increase in clamping pressure to 2.0 and 2.5 atm only prevented the frictional heat release between the joined parts since higher applied pressure hindered the possibility of upper adherend expansion and their mutual displacements under US vibrations (at a low USW duration of *t* = 600 ms). As a result, the USW lap joint thinning did not exceed 160 and 120 µm, respectively, (Table 3) after the USW processes when mode 2 was in use (*t* = 600 ms, *P* = 2.0 atm) and mode 3 (*t* = 600 ms, *P* = 2.5 atm). At the same time, the CFF thickness changed slightly relative to its initial value of ~250 µm (Table 4). Since the ED almost did not melt at such a short duration of US vibrations (Figure 6b,c), the formed USW lap joints were unreliable. They fractured at σ_UTS_ = 6–8 MPa (Figure 4) due to an adhesive delamination of one of the PEEK adherends (Figure 5b,c). In both cases, the EDs melted to a lesser extent than at the clamping pressure of 1.5 atm (mode 1) since a higher clamping pressure suppressed mutual displacements between the adherends, EDs, and the prepreg.

With increasing the USW duration to 850 ms, melting of the PEI binder in the prepreg actively developed due to frictional heating intensification. This resulted in USW lap joint thinning of 300 µm (Table 3) for mode 4 (*t* = 850 ms, *P* = 1.5 atm). The “excessive” polymer melted in the US-welding zone and was squeezed out beyond its boundaries (Figure 5d). At the same time, the thickness of the CFF remained about its initial value of ~250 μm (Table 4) with a variation in its thickness of ~80 μm (Figure 6d), maintaining the prepreg structural integrity. The reliable lap joint formation was confirmed by its high ultimate tensile strength of 37 MPa (Table 3).

The subsequent increase in clamping pressure to 2.0 and 2.5 atm was accompanied by greater squeezing out of the molten polymer from the prepreg and partially from the EDs. The USW lap joint thinning was about ~400 µm in both cases (Table 3). At the same time, the CFF thickness decreased at ~50 µm (Table 4). Visually, the CFF retained its structural integrity (Figure 6e for mode 5 at *t* = 850 ms and *P* = 2.0 atm; Figure 6f for mode 6 at *t* = 850 ms and *P* = 2.5 atm). However, its thickness variation slightly increased to 90–100 µm (Table 4). The ultimate tensile strength values were ~50 and ~40 MPa, respectively (Figure 4). Nevertheless, lateral tearing of the CFF was observed for mode 5, the fragments of which were squeezed out by the molten polymer flow from the welding zone (Figure 5e). The delamination of one PEEK adherend was accompanied by cohesive tearing of the CFF fragment. On the one hand, this increase in adhesion provided the maximum ultimate tensile strength of ~50 MPa. On the other hand, even the PEI-impregnated prepreg (some of which were extruded in the USW process) failed during the tensile test.

Because of the generally similar macrostructure (Figure 5d,f, respectively), the USW lap joint formed using mode 6 (*t* = 850 ms, *P* = 2.5 atm) and mode 4 failed similarly. Therefore, it is highly likely, the maximum implemented clamping pressure hindered the frictional heating development, which was also facilitated by the minimum clamping duration after US vibrations of 2000 ms. In this case, the distance between the joined PEEK adherends was ~190 µm, and the CFF thickness was ~200 µm at the center of the specimen after the USW process, according to the optical observation (Table 4).

Increasing the USW duration up to 1100 ms resulted in further squeezing out of the EDs and the prepreg due to enhanced frictional heating. For modes 7 and 8 (Figure 5g,h), the USW lap joint thinning was 700 µm, while it was 550 µm for mode 9 (Table 3). At the same time, the CFF thickness was significantly reduced to 110–160 µm (Table 4), and the variation in its value reached the initial level (Figure 5i). In other words, the CFF was locally completely damaged (Figure 6h), while locally, it was significantly “over-impregnated” with the molten polymer (Figure 6g–i). Thus, the CFF failure did not enable us to recommend modes 7–9 (at t ≥ 1100 ms) for the USW procedures developed for the PEEK plates with the PEI-impregnated CFF prepreg placed between them, despite the high ultimate tensile strength of 65–91 MPa.

Note that significant polymer remelting and mixing of the components, including the fragments of the fractured CFF, prevented the failure of the USW lap joints due to macrobending in all three cases (modes 7–9). Probably, the USW lap joint thinning down to 700 µm contributed to this phenomenon for modes 7 and 8.

By way of summarizing, the following remark on frictional heating is to be added. The USW duration was shown to mostly affect the frictional heating since mechanical vibrations are transformed into a mutual displacement of the US-weld components. The clamping pressure initially suppresses the mutual displacement and frictional heating. However, after melting the EDs and the prepreg, the former stimulates mass transfer and mutual mixing. Thus, the effect of the USW parameters is complex and cannot be characterized in an isolated way.

## 5. Neural Network Simulation

Determining the optimal USW conditions (combinations of the process parameters) from a limited number of experiments might be reduced to the problem of vector approximation of the mechanical properties of the USW lap joints in the multidimensional space of the USW modes [52]. Among the numerous mathematical methods designed to solve approximation problems, ANN simulation is considered one of the most appropriate.

Many papers reported some ANN applications and their advantages clearly [56,57]. They are most widely applied for solving problems of classification and recognition on large volumes of training samples (big data). However, in the case of a small amount of initial information (for instance, within nine experiments in the present study), a few issues arose [58,59], causing a high error in the simulation results in the approximation region and, especially outside the determination areas of the experimental parameters (forecast zones). 

**Figure 7:**
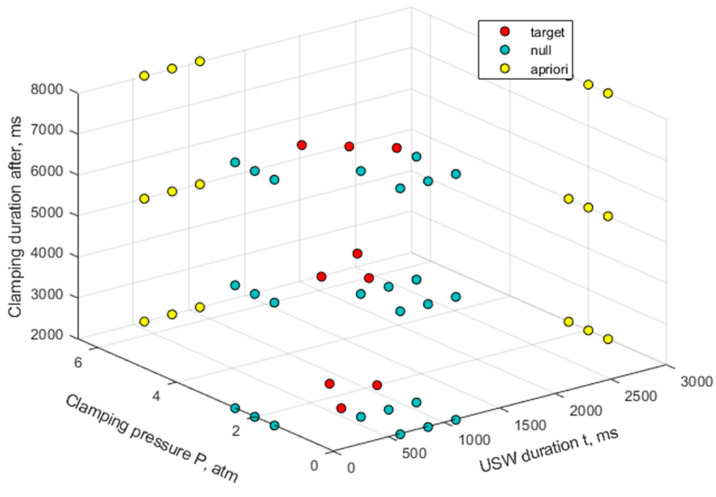
The distribution of the experimental parameters and selected priors for the USW results. The USW parameters used in the training sample: 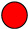 the experimental parameters; 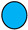 the parameters under which lap joints are not formed; 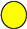 the parameters when the prepreg is fractured.

Previously, we proposed a solution based on increasing the sample size by adding a random noise and synthesizing several models [52]. However, there is another way to minimize simulation errors. It is based on adding a priori known data for the boundary values of the parameters to the experimental data. For example, it was known that any joint would not form (since zero pressure eliminates frictional heating) under conditions of zero-clamping pressure and arbitrary values of clamping duration after US vibrations [52]. In this case, the mechanical properties would have zero values, while the original structural characteristics of the joined parts would remain unchanged. Similar results would be obtained for any clamping pressure and negligible USW durations (*t* ≤ 1000 ms). Figure 7 presents the analysis results of the distribution of the experimental parameters and selected priors for which the USW results were known (the tabulated data values, shown in Figure 7, are presented in the Supplementary section, Table S6).

ANN simulation was carried out with the MathLab software package, which has the standard tools for the synthesis, training, and analysis of ANN. In addition, feedforward neural networks (FFNs) and radial basis function networks (RBFs) were implemented. The number of neurons in the hidden layer varied from four to eight, and the activation functions (linear, hyperbolic tangent, and logistic) were used.

By analogy with the technique described in [52], two-sided restrictions were applied, which determined the region of the optimal USW lap joint characteristics (Table 5) and the USW parameters studied in Section 4.

Among many developed models based on experimental and a priori data, the two were chosen. They provided the minimum acceptable root mean square approximation error (the bottom limit) and excluded excessive nonlinearity of approximation (due to overtraining of networks). The latter condition was the top limit.

Model 1. The FNNs were implemented with six neurons in the hidden layer and the activation functions: linear in the hidden layer and hyperbolic tangent in the output one. The training was carried out on a sample of the normalized data (with zero mean and unit variance). The sample was added with a synthesized data set obtained by a random spread of the normalized experimental data with the uniform distribution law [–0.05, 0.05].

Model 2. The RBN parameters were determined based on the analysis of the initial data: a spread of radial basis functions of 0.35 and the mean squared error goal of 0.0001. Then, training was done on a sample of the normalized data (with zero mean and unit variance).

**Figure 8:**
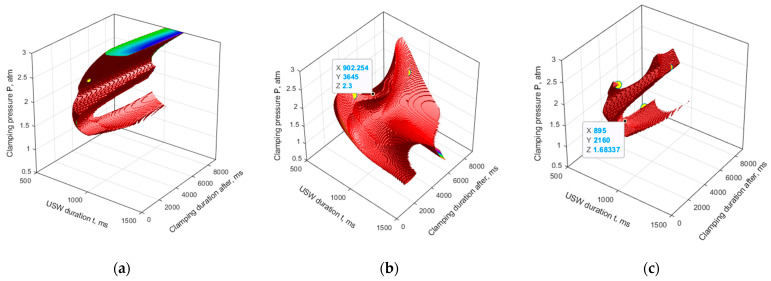
The areas of the optimal parameters gained due to the ANN simulation results: 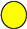 the experimental parameters corresponding to an optimal condition; (**a**) model 1, (**b**) model 2, (**c**) intersection of the areas.

The areas with the determined optimal parameters for both models are shown in Figure 8a,b. The models revealed the complex shape pattern and the ambivalence of the solution to the problem. However, the approach applied in this research with the use of a priori data enabled us to localize the areas.

To identify general patterns of the simulation results, crossing the areas of the optimal parameters was performed for both models (Figure 8c). The following parameters were chosen *t* = 900 ms, *P* = 1.7 atm, *τ* = 2.000 ms (marked in Figure 8c and designated as mode 10) for the model verification due to the following considerations:

1. The point was in the center of the bottom segment of a predicted area for the optimal parameters.
2. The point did not fall in the top segment. Instead, it referred to lower clamping pressures P. According to the authors, this had to minimize the damage of the melted prepreg during the USW.
3. The point was equidistant from two neighboring experimental ones.

However, the top segment of the “region of interest” in this setting remained uncovered. For this reason, we chose a point from this segment for subsequent verification, shown in Figure 8b (*t* = 900 ms, *P* = 2.3 atm, *τ* = 3600 ms; designated as mode 11). The point in the bottom segment (mode 10) was between modes 4 and 5, while another (mode 11) in the top segment was between modes 5 and 6.

## 6. Verification of the Neural Network Model

**Figure 9:**
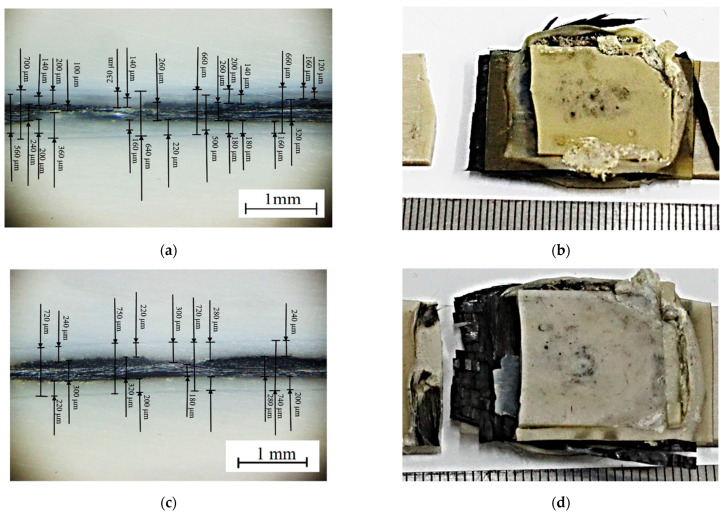
Optical images of the cross sections (**a**,**c**) and general views of the failed USW lap joints after the tensile tests (**b**,**d**) obtained by using modes 10 (**a**,**b**) and 11 (**c**,**d**).

Table 6 and Table 7 show the results of the mechanical tests of the USW lap joints obtained under optimal USW conditions. For mode 10, both ultimate tensile strength and elongation at break values were significantly higher than those for mode 4. However, they were almost identical to those for mode 5 (which did not fall into the optimal region). In this case, there was a noticeable USW joint thinning to 430 µm. This meant that the USW process was accompanied by high frictional heating and the plastic strains of both the prepreg and the ED (Table 6). According to Table 7, the CFF thickness remained almost at the initial level of ~270 µm, although the distance between the adherends decreased to 560 µm (according to the optically measured data, Figure 9a).

After the implementation of mode 11, both ultimate tensile strength and elongation at break values were lower, while the USW joint thinning was slightly greater (450 µm, according to Table 6). In general, the parameters of mode 11, given in Table 6, were almost identical to those for mode 6 (Table 3). The same could be stated about the dimensional characteristics given in Table 7 and evaluated optically (Figure 9c). The fracture pattern of the USW lap joints, obtained by using modes 6 and 11, could also be considered similar (Figure 9b,d).

Based on the results of the verification of ANN simulation data, mode 10 was chosen as the optimal mode, using which the USW lap joints were obtained for both tension and fatigue tests. Their typical stress–strain diagram (Figure S2) indicated that they exhibited a near-elastic strain behavior almost up to the fracture point (ε~1.9%), characterized by the brittle type. The latter was manifested through an abrupt stress drop exactly at reaching the ultimate strength. Table 8 presents the key mechanical properties.

Under quasi-static loads, the ability to be plastically strained for the PEEK plates was suppressed by the presence of the prepreg (the yield point of the USW lap joints was comparable to its tensile strength), causing the brittle fracture pattern. In addition, a significant difference in the elastic modulus values of the CFF and the PEEK plates (adherends) contributed to the development of both shear stresses and strains at the interlayer boundary. Thus, the strength properties of the USW lap joints depended on a level of adhesion between the prepreg and the PEEK adherends. Respectively, the fracture was induced by discontinuities at the interlayer boundaries.

Photographs of the USW lap joint surfaces, captured in the tensile tests, with superimposed longitudinal ε_YY_ strain fields under analysis (Figure S3). Up to the pre-fracture stage, the CFF retained its structural integrity, and its fragments did not protrude beyond the specimen contours (Figure S3a–c). Before the failure, on the contrary, numerous fractured fibers were observed, bordering the contour of the specimen gauge length, while the CFF was torn together with the PEEK adherends in the fracture zone (Figure S3d). This fact indicated a satisfactory level of their adhesion to the prepreg.

**Figure 10:**
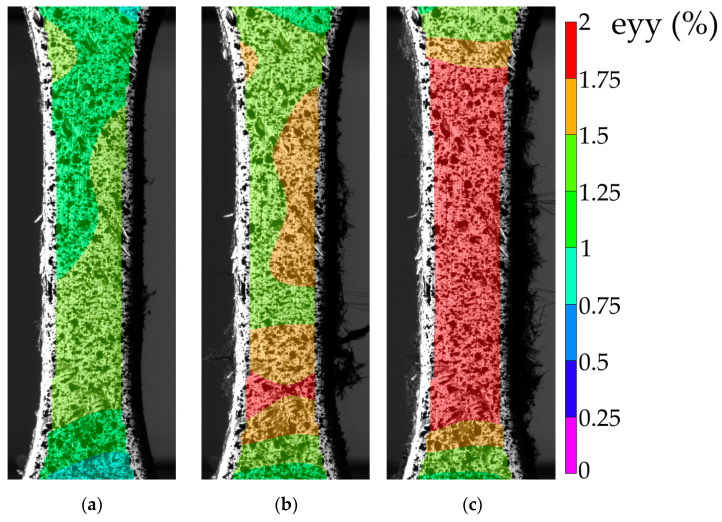
The strain development in the HCF tests: 1000 (**a**); 3000 (**b**); 7000 cycles (**c**).

Figure 10 shows the fields of ε_YY_ strain calculated from the HCF test results (*σ*_max_ = 50 MPa). Up to 1000 cycles (Figure 10a), ε_YY_ strains were uniformly distributed, and their maximum values did not exceed ~1.5%. At ~10,000 cycles (Figure 10b), a strain localization zone was formed at the bottom of the sample gauge length. Simultaneously, some fractured fibers of the CFF were observed on the side surfaces of the sample gauge length (mainly on the right). At the pre-fracture stage (Figure 10c), the CFF was damaged along the entire gauge length, which corresponded to an increase in ε_YY_ values up to ~2.0% over its entire surface.

**Figure 11:**
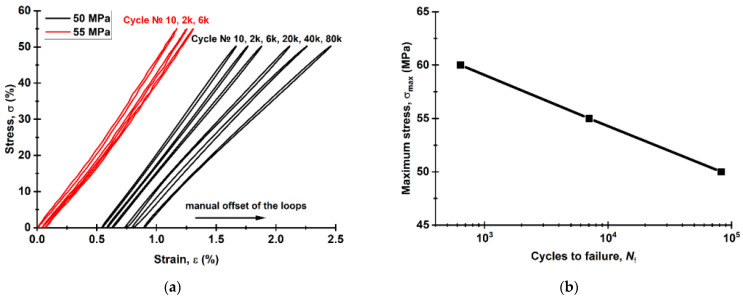
The fatigue test results for the USW lap joints: the mechanical hysteresis loops (**a**) and the fatigue curve (**b**).

It should be noted that the testing time of 82,000 cycles was achieved after 1.5 days at loading per cycle of 50 MPa. However, local discontinuities of the CFF were observed already after 6000 cycles. In order to reduce the testing duration, the load per cycle was increased to 60 MPa. After the load enhancement, the sample withstood about 638 cycles. This point is indicated on the fatigue curve (Figure 11b) as a separate test mode. Thus, the durability was assumed to be conditionally equal to 82,638 cycles for the load per cycle of 50 MPa. The fatigue curve was linear, which might indicate that the sample would have a durability comparable to 638 cycles at the maximum load per cycle of 60 MPa. 

The mechanical hysteresis loops, drawn with the DIC method, are shown in Figure 11a (black curves). The small area of the loops testifies to the low ductility of the samples. As the fatigue tests progressed, both loop shapes and their inclination angles slightly changed. This fact indicated that the proportion of the inelastic strains was minimal at the maximum load per cycle. At the same time, a fatigue failure was mainly due to damaging the CFF and lowering its adhesion to the PEEK binder.

**Figure 12:**
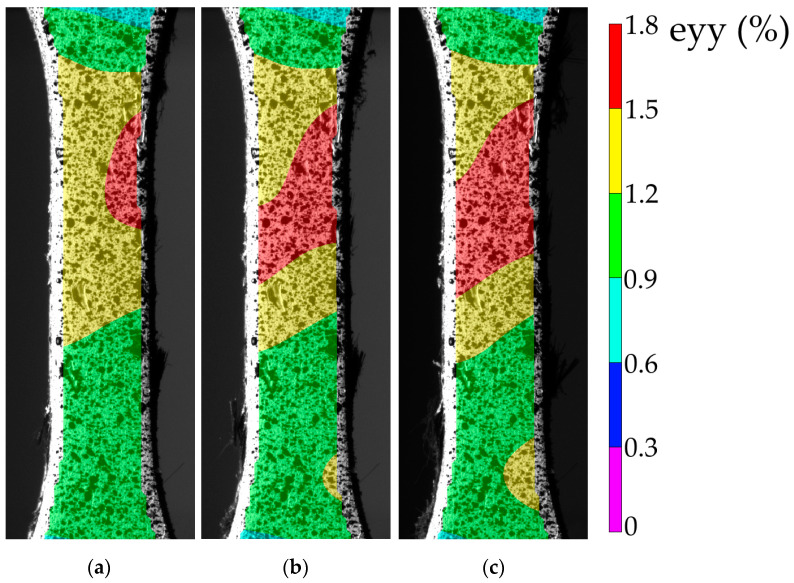
The strain development in the LCF tests: 1000 (**a**); 3000 (**b**); 7000 cycles (**c**).

For the LCF mode (σ_max_ = 55 MPa), a change in the hysteresis loop slope was observed when the operating time reached 2000 and then 6000 cycles, reflecting a decrease in the sample stiffness (Figure 11a). In contrast to the HCF mode, the upper half of the loop was tilted to the left (typically, it was tilted to the right, see black loops). The authors attributed the loss of the sample stiffness to the damage development mainly in the region of the interlayer boundary of the CFF and the PEEK adherends. This agrees well with the sample’s appearance during the fatigue tests (Figure 12). However, this stress level, despite the occurrence of local adhesive damage, did not result in a fast failure. The fatigue (Wöller) curve, shown in the semi-logarithmic coordinates in Figure 11b, possessed a linear pattern in the studied load range. The LCF-to-HCF transition corresponded to the range from 10^3^ to 10^4^ cycles. The endurance limit could not be identified in the framework of the tests carried out in this research. The graphs in Figure 11 indicate the reliable US-welding of the PEEK adherends and might be further used for establishing a correlation between the structure of the joints and their loss to resist cyclic loading.

For the LCF mode (σ_max_ = 55 MPa), the strain fields are shown in Figure 12. At the stress per cycle of 55 MPa, the strains were localized in the upper part of the sample already at 1000 cycles (Figure 12a). A gradual increase in the size of the localized strain area up to ~1.8% indicated the delamination development under the PEEK adherends (Figure 12b). Just before the fracture (Figure 12c), the strain localization extended to the entire cross-section but not to the entire area of the gauge length.

**Figure 13:**
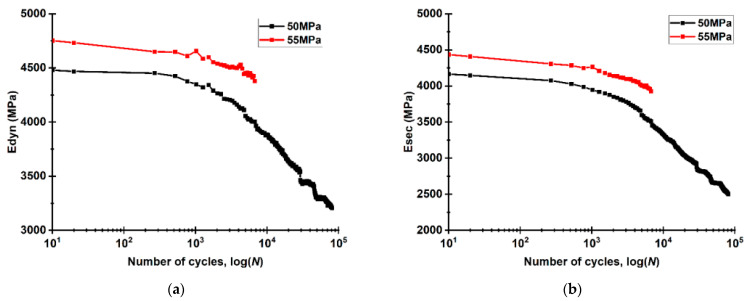
The changes in the dynamic characteristics of the USW lap joints in the fatigue tests: dynamic (**a**) and secant (**b**) moduli.

Using the DIC method, the parameters of the mechanical hysteresis loops were calculated for both LCF and HCF modes, namely, the dynamic and secant moduli, as well as the loop areas and the residual strain values [60]. Figure 13 shows that both secant and dynamic moduli were higher at the load per cycle of 55 MPa. For the HCF mode, the dynamic modulus decreased smoothly at ~6000 cycles (Figure 13a), while a downward trend appeared for the secant modulus already after ~1000 cycles (Figure 13b; black curve). In the HCF mode at the load per cycle of 55 MPa, both moduli reduced insignificantly at ~1000 cycles, after which the lowering rate increased, possessing an almost constant value starting from ~4000 cycles until a failure. The graphs in Figure 13 describe the evident indicators of losing the bearing capacity as the structure of the welds degrades.

It was assumed that the failure of the CFF and the USW lap joint was almost simultaneous in the HCF case. However, the fracture of the CFF occurred while maintaining the integrity of the PEEK adherends when reaching 6000 cycles. An accelerated decrease in the elastic modulus indicated a loss of stiffness and stretching, which was only possible if the CFF reinforcement was absent.

**Figure 14:**
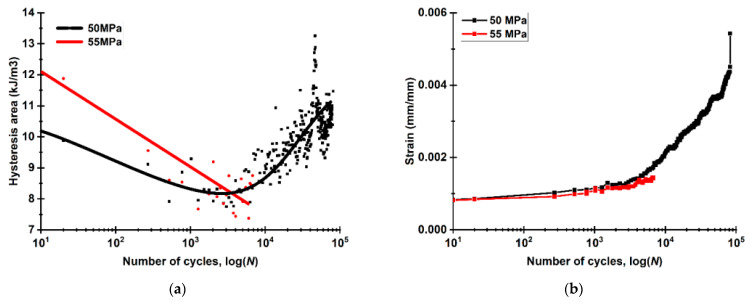
The strain behavior of the USW lap joints in the fatigue tests in terms of the mechanical hysteresis loop areas (**a**) and the residual strains (**b**).

Further, changes in both areas of the hysteresis loops, reflecting the mechanical energy loss (mainly due to dissipation), and the residual strains were drawn for both LCF and HCF modes. According to Figure 14a, there was a gradual decrease in the loop areas up to 6000 cycles for both modes. At the same time, the loop areas were almost equal at a level of about 8 kJ/m^3^. For the HCF case, the loop areas further increased up to 11 kJ/m^3^ by the failure point (Figure 14a). The scatter of the hysteresis loop area measurements was related to the specific features of the measuring technique [60]; however, it is not critical in terms of the revealed regularities.

Figure 14b shows the residual strain kinetics. At the initial loading stages (up to 6000 cycles), the residual strains enhanced insignificantly. However, at the next stage, there was an inflection and a subsequent non-linear increase in the residual strains (especially for the HCF case). By the failure point, their values reached 0.0055 mm/mm (0.55%), indicating damage to the CFF, which was not able to bear the load at these loading stages. This caused stretching of the USW lap joints.

## 7. Prospects (USW of Particulate and Laminated PEEK-CF Lap Joints)

This study was a further development of our previous research [52], both in terms of the formation of the USW lap joints from the neat PEEK adherends with the CFF interlayer. The current study has shown that it is necessary to impregnate the CFF with the PEI solution, at which both thermal and mechanical properties were comparable with those of the PEEK adherends in order to increase the strength characteristics. In addition, ANN simulation was further developed. This enabled us to determine the optimal USW parameters. As a result, the high strength characteristics were achieved, while the structural integrity of the CFF was maintained. The ANN was trained based on the results of the limited experimental sample with the additional expert characteristics of the materials considered by the authors.

Further, it was important to estimate the applicability of the results of ANN simulation for the development of the USW procedures for particulate or laminated composites. In response, the particulate composite samples were fabricated containing 30 wt.% CFs (MCFs; Tenax^®^-A, Teijin Carbon Europe GMBH) in the “PEEK770 MCF” matrix (Tenax^®^-A, Teijin Carbon Europe GMBH). They possessed a characteristic length of 200 μm at an aspect ratio of ~30. In addition, the “CF/PEEK770” laminated composites were made, consisting of 10 layers of bidirectional “ACM C285S” CFF at a ratio of 40 wt.% PEEK/60 wt.% CF. They were used as the adherends, while the ED films and the PEI-impregnated prepreg with the CFF were inserted in the same way as in the previous sections.

**Figure 15:**
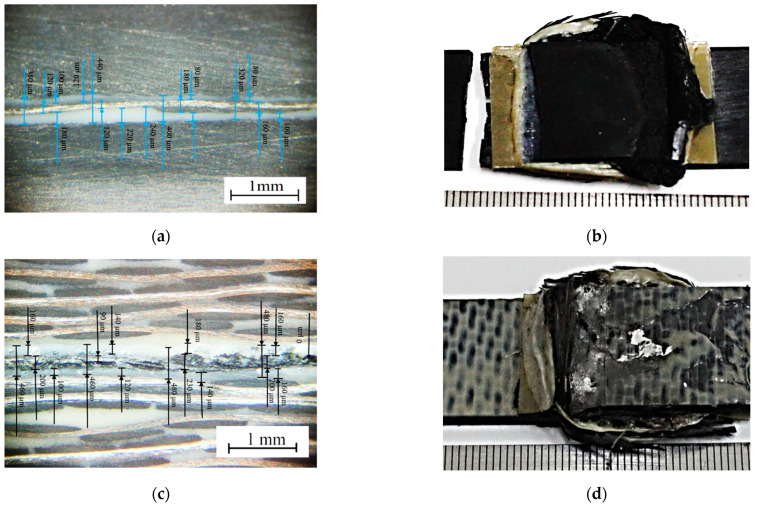
Optical images of the cross sections (**a**,**c**) and general views of the failed USW lap joints after the tensile tests (**b**,**d**), obtained with modes 12 (particulate composite adherends, (**a**,**b**)) and 13 (laminate composite adherends, (**c**,**d**)).

It should be noted that all attempts to form a USW lap joint at the “optimal” parameters established for the neat PEEK adherends (modes 10 and 11) failed for the CF-enforced PEEK ones (the melting of the weld components did not initiate at all). Nevertheless, experimentally, by increasing the USW duration (at constant both *P* and *τ* values), the USW lap joints were obtained for the particulate (mode 12) and laminate PEEK/CF (mode 13) composites. Their mechanical properties are presented in Table 9, and the dimensional characteristics, calculated from the optical images (Figure 15a,c), are reported in Table 10. Their strain–stress diagrams are shown in Figure S4. The strength properties were greater for the USW lap joint of the laminates. In this case, both elastic modulus and ultimate tensile strength values were maximal (Table 9), while their failure was developed by the interlayer shear mechanism (Figure 15d). So, the LSS value was estimated as LSS = 11.4 MPa. At the same time, the failure of the USW lap joint of the particulate composite occurred due to its macrobending (Figure 15b).

Although the USW lap joint of the particulate composite was formed at the longer USW duration of *t* = 1200 ms, the USW joint thinning was 460 µm, comparable to that for the neat adherends using mode 10. For the laminates, the Δ*h* value was lower (350 µm) at *t* = 1600 ms. At the same time, the CFF was noticeably thinned (Figure 15a) to 140 µm (Table 10). In the laminate case, the CFF retained its structural integrity, while the ED did completely melt (Figure 15c).

## 8. Conclusions and Outlook

The obtained results allowed us to draw the following conclusions.

- The proposed method of impregnating the CFF with the PEI solution was shown to be efficient for implementing the USW process for the neat PEEK adherends and allowed us to maintain the integrity of the CFF under US vibrations exposure (at the prepreg thickness of ~250 µm, which was comparable to that of the initial CFF). However, when USW the “PEEK–ED (PEEK)–prepreg (PEI impregnated CFF)–ED (PEEK)–PEEK” composites, the lower melting point and the higher melting flow index caused an extrusion of the molten PEI, which could be accompanied by damage to the CFF.
- By ANN simulation, based on the sample of the experimental data expanded with the expert data set, the optimal modes were determined for the USW lap joint of the “PEEK–ED (PEEK)–prepreg (PEI impregnated CFF)–ED (PEEK)–PEEK” composites. Furthermore, the experimental verification of the simulation results was carried out, which showed that mode 10 (*t* = 900 ms, *P* = 1.7 atm, *τ* = 2000 ms) ensured the high strength properties and preservation of the structural integrity of the CFF.
- It was shown that the “PEEK– CFF prepreg–PEEK” the USW lap joint could be fabricated by the “multi-spot” USW method using the optimal mode 10. It was capable of resisting the load per cycle of 50 MPa (the bottom HCF level).
- The USW mode, determined by ANN simulation for the neat adherends, did not provide joining both the particulate and laminated composite adherends with the CFF prepreg reinforcement. As a result, the USW lap joints were formed only by significantly increasing the USW durations *t* up to 1200 and 1600 ms, respectively. This occurred since the elastic energy transferred to the welding zone through the upper adherend.

The proposed approach for ANN simulation of the USW process can be implemented to determine the optimal USW modes for the particulate and PEEK/CF laminates, including those containing the CFF (prepreg) layer. However, a new training sample should be formed for this purpose, based on the experimental data and augmented with a priori results.

## Figures and Tables

**Table 1:** The combination of the USW parameters and their levels (according to the Taguchi table in L9 format for a three-factor experiment).

Mode/Experiment Number	Level/Factor		
	USW Duration (*t*), ms	Clamping Duration after US Vibrations (*τ*), ms	Clamping Pressure (*P*), atm
1	1/600	1/2000	1/1.5
2	1/600	2/5000	2/2.0
3	1/600	3/8000	3/2.5
4	2/850	1/5000	2/1.5
5	2/850	2/8000	3/2.0
6	2/850	3/2000	1/2.5
7	3/1100	1/8000	3/1.5
8	3/1100	2/2000	1/2.0
9	3/1100	3/5000	2/2.5

**Table 2:** The mechanical and dimensional properties of the USW lap joints obtained at *t* = 1000 ms, *P* = 1.5 atm, and τ = 5000 ms.

Binder	Ultimate Tensile Strength (σ_UTS_), MPa	Elongation at Break (ε), %	Elastic Modulus (E), MPa	USW Joint Thinning (Δ*h*), mm
PES	40.5 ± 1.5	1.8 ± 0.2	2034 ± 110	410 ± 20
PEI	50.1 ± 1.8	2.1 ± 0.2	2175 ± 90	290 ± 15

**Table 3:** The mechanical and dimensional characteristics of the USW lap joints for various USW parameters.

Mode Number	$\mathrm{Ultimate}\;\mathrm{Tensile}\;\mathrm{Strength}\;(\sigma_{UTS}),\;\mathrm{MPa}$	Elongation at Break (ε), %	Work of Strain (*A*), N·m	Elastic Modulus (*E*), MPa	USW Joint Thinning (Δ*h*), mm
1	16.1 ± 1.5	0.9 ± 0.1	7.6 ± 0.5	2014 ± 110	0.09 ± 0.03
2	5.6 ± 0.4	1.0 ± 0.1	3.4 ± 0.2	740 ± 68	0.16 ± 0.05
3	8.2 ± 0.6	1.7 ± 0.2	9.1 ± 0.7	1180 ± 102	0.12 ± 0.04
4	36.7 ± 1.8	2.1 ± 0.2	38.7 ± 2.5	2017 ± 90	0.30 ± 0.03
5	49.6 ± 3.4	2.9 ± 0.2	76.1 ± 4.4	2314 ± 115	0.40 ± 0.04
6	39.8 ± 1.9	2.2 ± 0.2	45.0 ± 3.2	2230 ± 121	0.40 ± 0.03
7	90.8 ± 4.1	7.4 ± 0.4	449.8 ± 15.7	2576 ± 130	0.70 ± 0.06
8	72.1 ± 3.5	4.4 ± 0.3	180.8 ± 10.4	2511 ± 123	0.70 ± 0.06
9	64.7 ± 3.2	3.6 ± 0.3	123.5 ± 9.3	2471 ± 119	0.55 ± 0.03

**Table 4:** The dimensional characteristics of the USW lap joints.

Mode Number	Initial Total Thickness of Lap Joined Parts, mm	USW Lap Joint Thickness, mm	Distance between PEEK Plates after USW, µm	CFF Thickness after USW, *d*_CF_, µm
	Contact		Optical (Photo)	
1	5.09	5.00	820 ± 40	350 ± 70
2	5.16	5.00	660 ± 80	270 ± 90
3	5.07	4.95	650 ± 50	260 ± 80
4	5.00	4.70	740 ± 60	250 ± 70
5	5.13	4.73	680 ± 120	190 ± 90
6	5.17	4.77	560 ± 60	200 ± 100
7	5.04	4.34	560 ± 60	160 ± 140
8	5.19	4.49	530 ± 70	110 ± 110
9	5.19	4.64	440 ± 60	160 ± 160

**Table 5:** Threshold values of the optimal USW lap joint characteristics.

Ultimate tensile strength (σ_UTS_), MPa	30 < σ_UTS_ < 60
Elongation at break (ε), mm	2.0 < ε < 3.5
USW lap joint thinning (Δ*d*), mm	0.15 < Δ*d <* 0.50
Distance between PEEK adherends after USW (*d*_adh_), µm	550 *< d_adh_* < 750
CFF thickness after USW (*d*_CF_), µm	170 < *d*_CF_ ≤ 350

**Table 6:** The mechanical and dimensional characteristics of the USW lap joints obtained with the parameters optimized by ANN simulation.

Mode Number	$\mathrm{Ultimate}\;\mathrm{Tensile}\;\mathrm{Strength}\;(\sigma_{UTS}),\;\mathrm{MPa}$	Elongation at Break (ε), %	Elastic Modulus (*E*), MPa	USW Joint Thinning (Δ*h*), mm
10 (900/1.7/2000)	47.1 ± 4.1	2.7 ± 0.2	2197 ± 109	0.43 ± 0.03
11 (900/2.3/3600)	39.9 ± 3.2	2.1 ± 0.2	2357 ± 121	0.45 ± 0.03

**Table 7:** The dimensional characteristics of the USW lap joints obtained with the parameters optimized by ANN simulation.

Mode Number	Initial Total Thickness of Lap Joined Parts, mm	USW Lap Joint Thickness, mm	Distance between PEEK Plates after USW, µm	CFF Thickness after USW, *d*_CF_, µm
	Contact		Optical (Photo)	
10	5.11	4.68	560 ± 60	270 ± 30
11	4.98	4.53	610 ± 130	240 ± 100

**Table 8:** The mechanical properties of the USW lap joint obtained with mode 10.

Ultimate Tensile Strength (σ_UTS_), MPa	Elastic Modulus (*E*), GPa	Elongation at Break (*ε*), %	Yield Point 0.2% (σ_0.2_), MPa
66.7 ± 4.3	3.9 ± 0.2	1.9 ± 0.1	64.4 ± 4.3

**Table 9:** The mechanical and dimensional properties of the USW lap joint obtained with modes 12, 13.

Mode Number	$\mathrm{Ultimate}\;\mathrm{Tensile}\;\mathrm{Strength}\;(\sigma_{UTS}),\;\mathrm{MPa}$	Elongation at Break (ε), %	Elastic Modulus (*E*), MPa	USW Joint Thinning (Δ*h*), mm
12 (1200/1.7/2000)	57.7 ± 4.5	1.5 ± 0.1	5613 ± 405	0.460 ± 0.03
13 (1600/1.7/2000)	84.5 ± 6.2	1.4 ± 0.1	9926 ± 524	0.350 ± 0.02

**Table 10:** The dimensional characteristics of the USW lap joints with the CFF prepreg between the adherends.

Mode Number	Initial Total Thickness of Lap Joined Parts, mm	USW Lap Joint Thickness, mm	Distance between PEEK Plates after USW, µm	CFF Thickness after USW, *d*_CF_, µm
	Contact		Optical (Photo)	
12 (1200/1.7/2000)	4.56	4.1	380 ± 60	140 ± 40
13 (1600/1.7/2000)	6.14	5.79	460 ± 20	185 ± 95

## Data Availability

The data presented in this study are available on request from the corresponding author. The data are not publicly available due to confidential disclosure reasons.

## Supplementary Materials

The following supporting information can be downloaded at: https://www.mdpi.com/article/10.3390/ma16052115/s1, Table S1: The influence levels of the studied factors on the ultimate tensile strength values.; Table S2: The influence levels of the studied factors on the elongation at break values; Table S3: The influence levels of the studied factors on the work of fracture values; Table S4: The influence levels of the studied factors on the USW joint thinning values; Figure S1: The dependences of the physical and mechanical characteristics of the USW lap joints on the parameter levels: ultimate tensile strength (a), elongation at break (b), work of strain (c), USW joint thinning (d); Table S5: The general influence levels of the studied factors on the USW lap joint characteristics; Figure S2. The strain–stress diagrams for the spot USW joints for fatigue tests; Figure S3: Photographs of the USW lap joint surfaces in the tensile tests: at the beginning stage (a); the strain localization stage (b); the pre-fracture stage (c); the fracture stage (d): Table S6. The USW parameters and a priori data values for the neural network simulation.
