# Supplementary material for: Ultrasonic Welding of PEEK Plates with CF Fabric Reinforcement—The Optimization of the Process by Neural Network Simulation
Source: Materials (Basel). 2023 Mar 6;16(5):2115. doi: 10.3390/ma16052115 (PMC10004364; doi:10.3390/ma16052115)
Supplement: Supplementary file 1 [file materials-16-02115-s001.zip › materials-2231293-supplementary.pdf]

materials

MDPI

Searching for the optimal USW mode for the PEI-impregnated prepreg by the Taguchi method and the data preparation process for the neural network simulation

The S/N parameters were determined by summing the obtained values at one factor level. A more significant difference between the S/N parameters at one level compared to that at another indicated a greater influence of the analyzed factor (Tables S1–S4). This variation was classified as the Delta parameter (or L_{max}–L_{min}, i.e. the difference in the S/N parameters between the maximum and minimum factor levels). This value reflected the parameter contribution to the change in the physical and mechanical characteristics during the transition from one level to another. As a result, it was determined which factor changed in a greater range of the values, causing a more noticeable variation in the physical and mechanical characteristics.

Table S1. The influence levels of the studied factors on the ultimate tensile strength values.

Levels	USW duration (*t*), ms	Clamping duration after US vibrations (*τ*), ms	Clamping pressure (*P*), atm
1	19.12551837	31.53051859	31.09762809
2	32.40020542	28.67736645	27.49172248
3	37.51283596	28.8306747	30.44920918
Delta	18.38731759	2.853152137	3.605905606

Table S2. The influence levels of the studied factors on the elongation at break values.

Levels	USW duration (*t*), ms	Clamping duration after US vibrations (*τ*), ms	Clamping pressure (*P*), atm
1	1.197012	7.637957	6.267452
2	7.5136	7.372338	5.856812
3	13.79325	7.493563	10.37959
Delta	12.59623	0.265619	4.522781

Table S3. The influence levels of the studied factors on the work of strain values.

Levels	USW duration (*t*), ms	Clamping duration after US vibrations (*τ*), ms	Clamping pressure (*P*), atm
1	15.80889268	34.14362672	31.94149688
2	34.1487209	31.13374667	28.07237893
3	46.67923223	31.35947242	36.62297
Delta	30.87033955	3.009880056	8.55059107

Table S4. The influence levels of the studied factors on the USW joint thinning values.

Levels	USW duration (*t*), ms	Clamping duration after US vibrations (*τ*), ms	Clamping pressure (*P*), atm
1	18.41637508	11.49025464	10.65732973
2	8.791725084	8.991479907	10.52264049
3	3.79627487	10.52264049	9.824404817
Delta	14.62010021	2.498774732	0.832924911

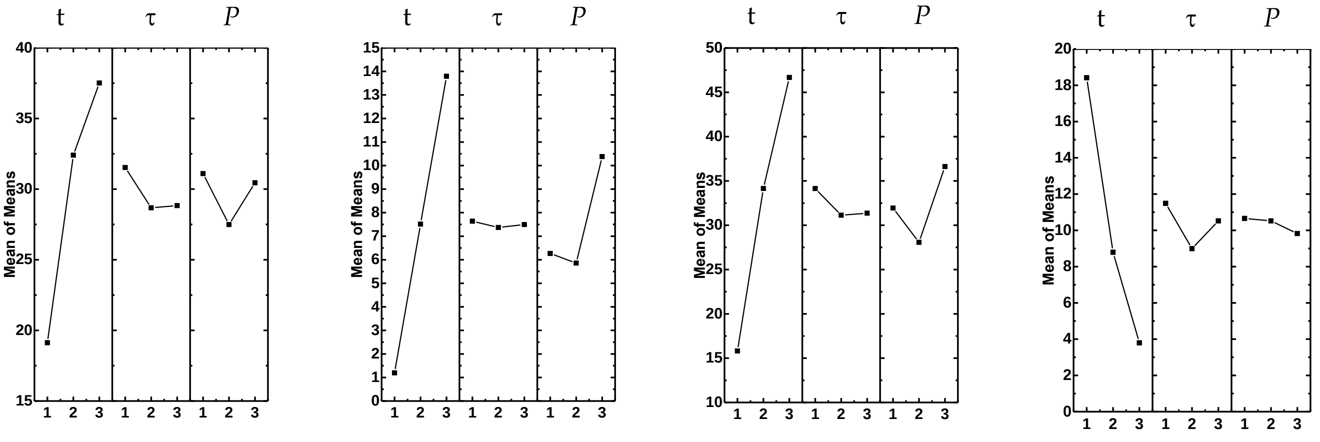

(**a**) (**b**) (**c**) (**d**)

Figure S1. The dependences of the physical and mechanical characteristics of the USW lap joints on the parameter levels: ultimate tensile strength (**a**), elongation at break (**b**), work of strain (**c**), USW joint thinning (**d**).

Based on the obtained results, the factors were further ranked, and their optimal values were determined according to the Taguchi method. A scoring system was summarized in Table S5. The factor that had the greatest impact was marked with the minimum number. It was determined that the USW duration *t* made the greatest contribution to the increase in the strength characteristics of the USW lap joints (its optimal value was 1100 ms.). The clamping pressure *P* affected to a lesser extent (the optimal *P* value was 1.5 atm). The clamping duration after US vibrations *τ* had a minimal effect, so it was decided to use its maximum value of 8000 ms for further research.

Table S5. The general influence levels of the studied factors on the USW lap joint characteristics.

Characteristic	USW duration (t), ms	Clamping duration after US vibrations (τ), ms	Clamping pressure (P), atm
Ultimate tensile strength (σUTS), MPa	1	3	2
Elongation at break (E), %	1	3	2
Work of fracture (A), N·m	1	3	2
USW joint thinning (Δh), mm	1	2	3
Total	4	11	9
Optimal value	1100	8000	1.5

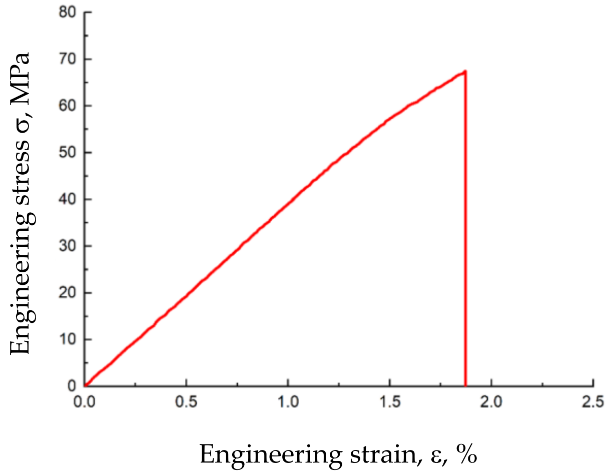

Figure S2. The strain–stress diagrams for the spot USW joints for fatigue tests

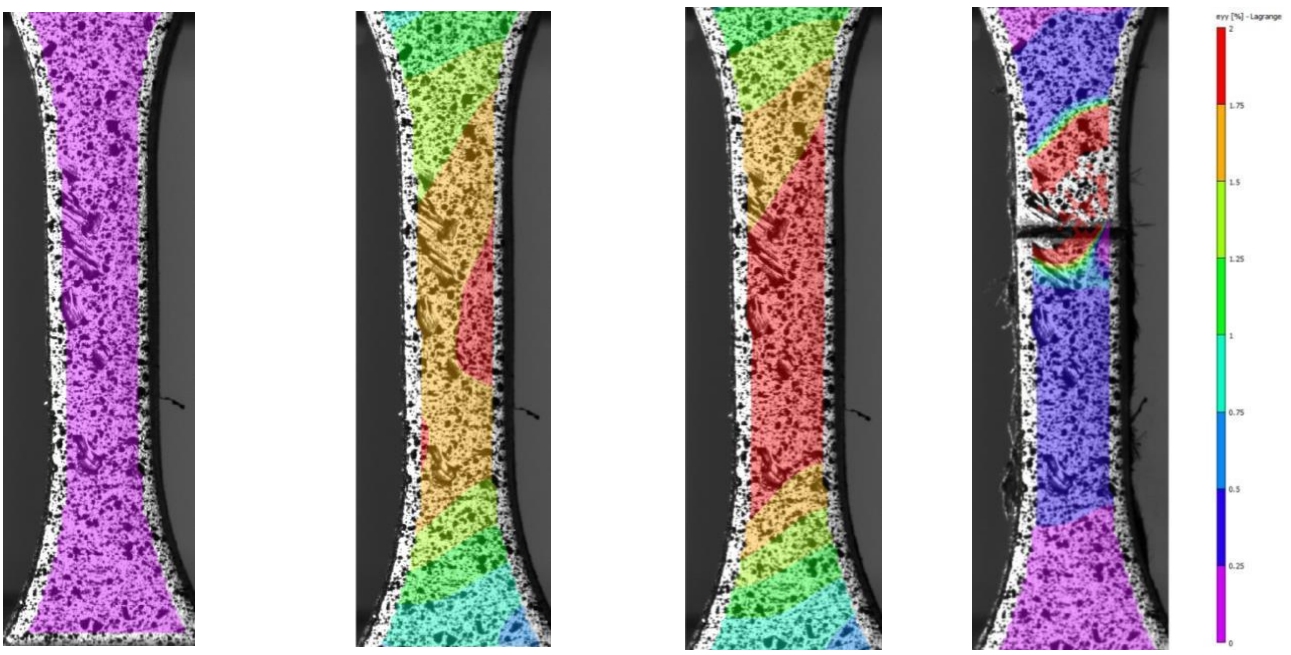

(**a**) (**b**) (**c**) (**d**)

Figure S3. Photographs of the USW lap joint surfaces in the tensile tests: at the beginning stage (**a**); the strain localization stage (**b**); the pre-fracture stage (**c**); the fracture stage (**d**).

Table S6. The USW parameters and a priori data values for the neural network simulation.

Virtual experiment number	USW duration (t), ms	Clamping duration after US vibrations (τ), ms	Clamping pressure, (P), atm	Ultimate tensile strength (σUTS), MPa	Elongation at break (ε), %	USW joint thinning (Δ*h*), mm	Distance between PEEK adherends after USW, μm	Fabric thickness after USW, dCF, μm
1	0	2000	1.5	0	0	0	750	250
2	0	5000	1.5	0	0	0	750	250
3	0	8000	1.5	0	0	0	750	250
4	0	2000	2.0	0	0	0	750	250
5	0	5000	2.0	0	0	0	750	250
6	0	8000	2.0	0	0	0	750	250
7	0	2000	2.5	0	0	0	750	250
8	0	5000	2.5	0	0	0	750	250
9	0	8000	2.5	0	0	0	750	250
10	600	2000	0	0	0	0	750	250
11	600	2000	1.0	0	0	0	750	250
12	600	5000	0	0	0	0	750	250
13	600	5000	1.0	0	0	0	750	250
14	600	8000	0	0	0	0	750	250
15	600	8000	1.0	0	0	0	750	250
16	850	2000	0	0	0	0	750	250
17	850	2000	1.0	0	0	0	750	250
18	850	5000	0	0	0	0	750	250
19	850	5000	1.0	0	0	0	750	250
20	850	8000	0	0	0	0	750	250
21	850	8000	0	0	0	0	750	250
22	1100	2000	0	0	0	0	750	250
23	1100	2000	1.0	0	0	0	750	250
24	1100	5000	0	0	0	0	750	250
25	1100	5000	1.0	0	0	0	750	250
26	1100	8000	0	0	0	0	750	250
27	1100	8000	1.0	0	0	0	750	250
28	3000	2000	1.5	90	7.5	800	400	50
29	3000	5000	1.5	90	7.5	800	400	50
30	3000	8000	1.5	90	7.5	800	400	50
31	3000	2000	2.0	90	7.5	800	400	50
32	3000	5000	2.0	90	7.5	800	400	50
33	3000	8000	2.0	90	7.5	800	400	50

34	3000	2000	2.5	90	7.5	800	400	50
35	3000	5000	2.5	90	7.5	800	400	50
36	3000	8000	2.5	90	7.5	800	400	50
37	600	2000	6.5	2	0.5	50	750	250
38	600	5000	6.5	2	0.5	50	750	250
39	600	8000	6.5	2	0.5	50	750	250
40	850	2000	6.5	2	0.5	50	750	250
41	850	5000	6.5	2	0.5	50	750	250
42	850	8000	6.5	2	0.5	50	750	250
43	1100	2000	6.5	3	0.7	50	750	250
44	1100	5000	6.5	3	0.7	50	750	250
45	1100	8000	6.5	3	0.7	50	750	250
